# Acidic pH Reduces Fluconazole Susceptibility in *Cryptococcus neoformans* by Altering Iron Uptake and Enhancing Ergosterol Biosynthesis

**DOI:** 10.4014/jmb.2504.04007

**Published:** 2025-05-27

**Authors:** Donghyeun Kim, Junghum Shin, Yong-Joon Cho, James W. Kronstad, Won Hee Jung

**Affiliations:** 1 Department of Systems Biotechnology, Chung-Ang University, Anseong 17546, Republic of Korea; 2 Department of Molecular Bioscience, Kangwon National University, Chuncheon 24341, Republic of Korea; 3 Multidimensional Genomics Research Center, Kangwon National University, Chuncheon 24341, Republic of Korea; 4 Michael Smith Laboratories, Department of Microbiology & Immunology, University of British Columbia, Vancouver, BC, V6T 1Z4, Canada

**Keywords:** Acidic pH, antifungal susceptibility, *Cryptococcus neoformans*, ergosterol, fluconazole, iron uptake

## Abstract

The opportunistic fungal pathogen *Cryptococcus neoformans* encounters diverse environmental pH conditions within the host. Hence, the ability to adapt to different pH levels plays a key role in survival and pathogenesis, although a full understanding of adaptation has yet to be achieved. In this study, we investigated how environmental pH influences antifungal drug susceptibility and iron uptake in *C. neoformans*. We found that acidic conditions significantly reduced the susceptibility of *C. neoformans* to the antifungal drug fluconazole. Moreover, iron acquisition in *C. neoformans* was independent of the high-affinity iron uptake system under acidic conditions, and lower pH increased the levels of intracellular iron, ergosterol, and heme, potentially accounting for the reduced susceptibility of the fungus to fluconazole. Transcriptome analysis further elucidated the mechanisms underlying the pH-dependent shift in iron uptake and antifungal susceptibility in *C. neoformans*. Overall, our findings highlight the importance of environmental pH in the physiology and pathogenesis of *C. neoformans* and provide insights to support the development of novel treatments for cryptococcosis.

## Introduction

The basidiomycete *Cryptococcus neoformans* is an encapsulated, opportunistic fungal pathogen that can cause life-threatening infections, primarily in immunocompromised individuals, such as patients with acquired immune deficiency syndrome (AIDS), organ transplant recipients, and patients undergoing immunosuppressive therapy [1, 2]. The prevalence of cryptococcal meningitis is particularly high among patients with human immunodeficiency virus (HIV) infection, with 180,000 deaths and up to 68% of HIV-related cases reported annually worldwide [1]. Despite advancements in antifungal therapy, cryptococcal meningitis remains a major global health threat, particularly in resource-limited countries where access to diagnostic tools and effective treatments is often limited [3].

The pathogenesis of *C. neoformans* involves several virulence factors that are required for fungal survival and disease progression within the host environment. Key virulence factors include the synthesis of a polysaccharide capsule, production of melanin, and the ability to grow at 37°C. The capsule, primarily composed of glucuronoxylomannan (GXM), is the predominant virulence factor. It protects the fungus from phagocytosis and modulates host immune responses. Melanin, synthesized by the enzyme laccase, protects against oxidative stress and contributes to the survival of the fungus within the host. The ability of *C. neoformans* to grow at a human body temperature of 37°C allows the fungus to proliferate and disseminate within the host body [4, 5].

Antifungal drugs, including amphotericin B, flucytosine, and azoles such as fluconazole, are typically used to treat cryptococcosis. Amphotericin B, belonging to the polyene family, directly binds to ergosterol, an essential constituent of the fungal cell membrane, thereby inducing pore formation, disrupting membrane integrity, and eventually causing cell death [6]. Flucytosine is a pyrimidine analog that inhibits DNA and RNA synthesis. It is often used in combination with amphotericin B [7]. Fluconazole, an azole, inhibits lanosterol 14α-demethylase (Erg11), a key enzyme in the ergosterol biosynthesis pathway. Ergosterol is an essential component of the fungal cell membrane. It maintains membrane fluidity and permeability. Inhibition of ergosterol synthesis can disrupt membrane integrity and functions and impair fungal growth [8, 9]. Fluconazole has several advantages, including its oral bioavailability, excellent central nervous system (CNS) penetration, and relatively low cost; therefore, it is often used as a first-line agent for maintenance therapy in patients with cryptococcal meningitis [10-12]. However, its efficacy can be limited by several factors, including the development of antifungal resistance and the presence of drug–drug interactions [13, 14]. In addition, environmental factors within the host can influence the susceptibility of *C. neoformans* to fluconazole [2, 15]. Therefore, understanding these factors is crucial for optimizing antifungal therapy and improving clinical outcomes.

Iron is essential for all living organisms, including fungi. It is necessary for numerous cellular processes, including respiration and DNA synthesis. *C. neoformans* is not an exception; it requires iron to synthesize essential molecules, such as heme, which is a component of cytochrome enzymes involved in the electron transport chain and acts as a cofactor for Erg11 activity. Iron acquisition is therefore crucial for the survival of *C. neoformans* within the host. *C. neoformans* employs several mechanisms to acquire iron from its environment. Under iron-limiting conditions, *C. neoformans* utilizes a high-affinity reductive iron uptake system involving the Cfo1 and Cft1 proteins. Ferric iron (Fe^3+^) becomes reduced to ferrous iron (Fe^2+^) by cell surface reductases encoded by *FRE* genes. In the cell membrane, Cfo1, a ferroxidase, oxidizes Fe^2+^ to Fe^3+^, which is then transported into the cytoplasm by the iron permease Cft1. This high-affinity reductive iron uptake system is essential for the survival and virulence of *C. neoformans* within the mammalian host environment [16-18]. Furthermore, studies have revealed that iron availability can influence the susceptibility of *C. neoformans* to antifungal drugs. For instance, in our previous study, deficiency in iron uptake and homeostasis significantly increased the susceptibility of *C. neoformans* to azole antifungal drugs [19]. Similarly, in another study, iron uptake and homeostasis contributed to azole susceptibility in *Candida albicans* [20]. These findings suggest that iron uptake and homeostasis play a key role in modulating the response of *C. neoformans* and other major fungal pathogens to antifungal therapy.

Pathogenic fungi encounter diverse environmental pH conditions. Adaptation to pH fluctuations therefore plays a key role in their survival, particularly within the host environment [21, 22]. *C. neoformans* has adapted to a broad range of pH conditions, from acidic to alkaline. Several studies have demonstrated that environmental pH levels can affect various aspects of the physiology of *C. neoformans*, including capsule production [23-25]. The pH of the host microenvironment can vary depending on the tissue type and the presence of inflammation. For example, inflamed tissues often exhibit acidic microenvironments because of increased metabolic activity and lactic acid production [26-28].

Given the importance of iron and pH in regulating fungal growth and virulence, we hypothesized that environmental pH could influence the susceptibility of *C. neoformans* to fluconazole by modulating iron uptake and ergosterol biosynthesis. In particular, we reasoned that acidic pH may alter iron availability and uptake, resulting in changes in ergosterol synthesis and consequently altering susceptibility to fluconazole. To test this hypothesis, we investigated the effects of different pH conditions on the susceptibility of *C. neoformans* to fluconazole, focusing on the interplay between iron uptake, heme biosynthesis, ergosterol production, and the expression of key *ERG* genes, in addition to the alteration of the transcriptome under different pH conditions. Indeed, we found that environmental pH modulates iron homeostasis and the expression of *ERG* genes, and consequently regulates ergosterol production to influence susceptibility to fluconazole. Our results will help to understand the pathogenesis of cryptococcosis and optimize antifungal treatment strategies.

## Materials and Methods

### Strains and Growth Conditions

*C. neoformans* H99 was used as the wild-type (WT) strain. The *cfo1* and *cft1* mutants used in this study were constructed previously [16, 17]. The fungal cells were routinely cultured in yeast extract peptone medium supplemented with 2% glucose (YPD) at 30°C. To grow the cells under iron-limiting conditions, low-iron medium (LIM) supplemented with 100 μM of bathophenanthrolinedisulfonic acid (BPS, Santa Cruz Biotechnology, USA) and adjusted to pH 7.0 with 3-(N-morpholino)-propanesulfonic acid (MOPS) was prepared and used as described previously [19]. Iron-replete medium was prepared by adding a final concentration of 100 μM of FeCl_3_, hemin, or ferroxamine to LIM. When necessary, the pH of each medium was adjusted to 5.0 with 5.8 M HCl.

### Construction of the Strain Expressing Cfo1–FLAG

To construct the Cfo1–FLAG fusion protein, *CFO1* was amplified by PCR using the primers Cfo1_BamHI_F and Cfo1_BamHI_R with WT genomic DNA. Amplified DNA fragments were digested with BamHI or SpeI and cloned into the plasmid pWH109 carrying the 3× FLAG sequence, the *GAL7* terminator, and the nourseothricin acetyltransferase gene [29]. The resulting plasmid pWH169 was linearized using HindIII and introduced into the WT strain by biolistic transformation. Positive transformants were confirmed by PCR.

### Antifungal Susceptibility Testing

The minimal inhibitory concentration (MIC) of fluconazole for *C. neoformans* was determined according to CLSI M27-A318 [30]. RPMI 1640 medium (Sigma, Germany) with L-glutamine and 2% glucose was prepared, and its pH was adjusted to 7.0 and 5.0 with MOPS and 5.8 M HCl, respectively. Fluconazole was serially diluted to a final concentration of 0.125–64 μg/ml and added to each well of a 96-well plate containing RPMI medium. *C. neoformans* cells pre-grown in YPD overnight at 30°C were inoculated into each well of the plate. The wells without the antifungal drug were used as positive controls, while those without cells were used as negative controls. MICs were determined at 48 h. To analyze antifungal susceptibility on solid LIM, *C. neoformans* cells pre-grown in YPD overnight at 30°C were harvested and then reincubated in LIM overnight at 30°C to deplete intracellular iron. The precultured cells were serially diluted 10-fold (1.0 × 10^5^ to 1.0 × 10 cells) and spotted on solid LIM (pH 7.0 or 5.0) with or without fluconazole (Sigma). The plates were incubated at 30°C for 3 days and then photographed.

### Western Blot Analysis

For western blot analysis, the fungal cells were harvested by centrifugation and resuspended in a protein extraction buffer consisting of 50 mM HEPES KOH (pH 7.5), 140 mM NaCl, 1 mM EDTA, 1% Triton X-100, 0.1%Na deoxycholate, 1 mM PMSF, and a protease inhibitor cocktail (Sigma). Cell lysates were prepared by bead-beating, and the protein concentration was determined using the Bradford assay [31]. In total, 20 μg of protein per sample was separated on a sodium dodecyl sulfate–polyacrylamide gel and transferred onto a nitrocellulose membrane (Sigma). Protein detection was performed using an anti-FLAG (Abcam, UK) rabbit polyclonal antibody. A mouse anti-rabbit IgG horseradish peroxidase conjugate (Santa Cruz, USA) was used as a secondary antibody, followed by visualization via chemiluminescence.

### Quantitative Real-Time PCR (qPCR)

The primers for real-time PCR were designed using Primer Express software 3.0 (Applied Biosystems) and are listed in Table S1. Total RNA was extracted using the RiboPure-Yeast Kit (Ambion), and cDNA was synthesized using the RevertAid First Strand cDNA Synthesis Kit (Fermentas). Relative gene expression was quantified using a 7500 system (Applied Biosystems) based on the 2^−ΔΔCT^ method. The translation elongation factor 2 gene (*TEF2*)(CNAG_00044) was used as a reference.

### RNA extraction, RNA Sequencing, and Data Analysis

The cells were grown overnight in YPD, harvested, and incubated overnight in YNB LIM (pH 7.0). The cells were then harvested and incubated in YNB LIM (pH 7.0 or 5.0) at 1 × 10^7^ cells/ml for 6 h. The cells were harvested by centrifugation, and total RNA was extracted using the RNeasy Mini Kit following the manufacturer’s instructions. Contaminating DNA was eliminated using RNase-free DNase (Invitrogen, USA). The quality of RNA was evaluated using a TapeStation RNA ScreenTape (Agilent Technologies, USA). Libraries for RNA sequencing were prepared with high-quality RNA (RNA integrity value > 7.0) using the TruSeq Stranded mRNA Sample Prep Kit (Illumina, USA) following the manufacturer’s protocol. The libraries were submitted to an Illumina HiSeq2500 platform, and 100-bp paired-end reads were generated and sequenced. Quality-filtered reads were mapped to the reference genome sequence (http://www.broadinstitute.org/annotation/genome/cryptococcus_neoformans/MultiHome.html) using CLC Genomics Workbench 4.0 (CLC Bio). Mapped reads were counted using featureCounts in Subread package v1.4.3, and relative transcript abundance was normalized to TPM [32]. The data were deposited in Gene Express Omnibus (GEO) under the accession number GSE291861.

### Inductively Coupled Plasma–Atomic Emission Spectroscopy (ICP–AES)

Total intracellular iron, zinc, copper, and manganese levels were determined by ICP–AES, as described previously [19]. In brief, the cells were grown overnight in YPD, transferred to YNB LIM (pH 7.0), and incubated overnight to deplete iron. Subsequently, the cells were harvested and incubated in YNB LIM (pH 7.0 or 5.0) at 1 × 10^7^ cells/ml for 6 h. The cells were then harvested, washed three times with low-iron water, and lyophilized. In total, 0.15 g of cell biomass was digested with 3 ml of H_2_O_2_ and 5 ml of HNO_3_ using the microwave digestion system START D (Milestone, Italy). ICP–AES was performed using an Optima 5300 DV system (PerkinElmer, USA). Scaling and normalization were performed based on the total cell mass.

### Determination of Intracellular Ergosterol Concentrations

Intracellular ergosterol concentrations in *C. neoformans* were measured as described previously [33]. In brief, *C. neoformans* cells were grown overnight in YPD, harvested, transferred to YNB LIM (pH 7.0), and incubated overnight. The cells were then harvested and incubated in YNB LIM (pH 7.0 or 5.0) with or without 100 μM FeCl_3_ at 1 × 10^7^ cells/ml for 16 h. The cells were harvested by centrifugation at 2,700 rpm for 5 min and washed with water. Subsequently, the cells were resuspended in 3 ml of 25% alcoholic potassium hydroxide solution and vortexed for 1 min. The cell suspensions were incubated in a water bath at 85°C for 1 h and cooled to room temperature. Following this, 1 mL of water and 3 mL of *n*-heptane were added to the cell suspensions and vortexed for 3 min. The heptane layer was transferred to a clean tube and kept at −20°C for upto 24 h. The sterol extract was diluted five-fold in 100% EtOH and spectrophotometrically scanned between 240 and 300 nm wavelengths.

### Determination of Intracellular Heme Levels

Intracellular heme levels were determined by colorimetric analysis using the BioVision Hemin Assay Kit (BioVision, USA). In brief, WT cells were grown overnight in YPD, transferred to YNB LIM (pH 7.0), and then incubated overnight to deplete iron. The precultured cells were reinoculated in YNB LIM (pH 7.0 or 5.0) at 1 × 10^7^ cells/ml for 6 h, washed twice with PBS, and resuspended in lysis buffer containing 10 mM Tris–HCl (pH 8.0) and 150 mM sodium chloride. The cells were lysed using a bead beater and centrifuged at 13,000 rpm for 3 min at 4°C. The supernatant was used to quantify heme levels according to the manufacturer’s instructions.

## Results

### Fluconazole Susceptibility Is Reduced at Acidic pH

How environmental pH influences the antifungal susceptibility of *C. neoformans* remains largely unknown. Therefore, we first determined the MIC of fluconazole at different pH levels. We chose fluconazole because it is considered a preferable broad-spectrum azole antifungal drug for treating cryptococcosis [11]. Moreover, as our previous study revealed that the deletion of *CFO1* increases fluconazole susceptibility, we included mutant strains lacking *CFO1* or *CFT1* to understand how the high-affinity iron uptake system affects the susceptibility to fluconazole at different pH levels [19].

**Fig. 1:**
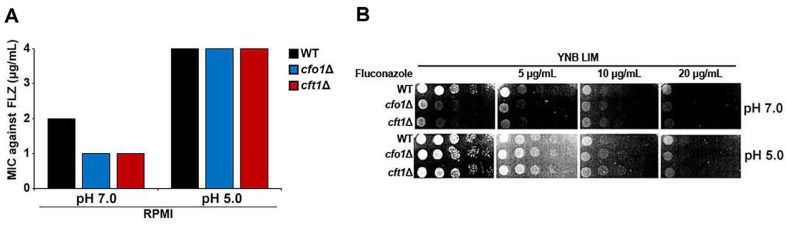
Susceptibility of *C. neoformans* to fluconazole at different pH levels. (**A**) MICs of *C. neoformans* WT, the *cfo1* mutant (*cfo1*Δ), and the *cft1* mutant (*cft1*Δ) were determined in RPMI media at pH 7.0 and 5.0. (**B**) Ten-fold serial dilutions of cells (starting at 10^5^ cells) were spotted onto solid LIM (pH 7.0 or 5.0). Plates were incubated at 30°C for 2 days and photographed. All experiments were performed three times; the representative results are presented.

The overall MICs for all strains (WT, *cfo1* mutant, and *cft1* mutant) were noticeably lower at pH 7.0 than at pH 5.0. These findings suggest that *C. neoformans* is generally less susceptible to fluconazole at acidic pH than at neutral or near-neutral pH, regardless of the strain background (Fig. 1A). Moreover, at pH 7.0, the MIC for the WT strain was higher than for the *cfo1* or *cft1* mutants, confirming our previous findings that the strains lacking high-affinity reductive iron uptake exhibit increased susceptibility to fluconazole at neutral pH [19]. The *cfo1* or *cft1* mutants may experience iron deprivation and thus exhibit reduced ergosterol biosynthesis and mitochondrial functions, resulting in increased fluconazole susceptibility. In contrast, at pH 5.0, all three strains (WT, *cfo1* mutant, and *cft1* mutant) exhibited similarly high MICs, implying that the loss of either *CFO1* or *CFT1* does not further increase the susceptibility of *C. neoformans* to fluconazole under acidic conditions. An explanation is that *C. neoformans* may rely on a different or additional iron uptake pathway not involving *CFO1* or *CFT1* under low pH conditions, allowing it to maintain ergosterol biosynthesis.

We also performed a growth assay on solid low-iron media containing different concentrations of fluconazole at different pH levels. At pH 7.0, the WT strain grew more robustly than the *cfo1* and *cft1* mutants in the presence of increasing fluconazole concentrations (Fig. 1B). However, in the presence of fluconazole, all strains exhibited noticeably better growth at pH 5.0 than at pH 7.0. Moreover, *C. neoformans* cells were less inhibited by fluconazole under acidic conditions. The differences in the growth of the WT strain and the *cfo1* and *cft1* mutants were less pronounced at pH 5.0. The mutants did not appear to be substantially more sensitive than the WT strain under acidic conditions, similar to the findings of our MIC assay. Overall, our data suggest that *C. neoformans* bypasses the high-affinity iron uptake system composed of Cfo1 and Cft1, and can use an unknown alternative iron uptake system under acidic conditions.

### The Expression of the High-Affinity Iron Uptake System Is Downregulated by Acidic pH

**Fig. 2:**
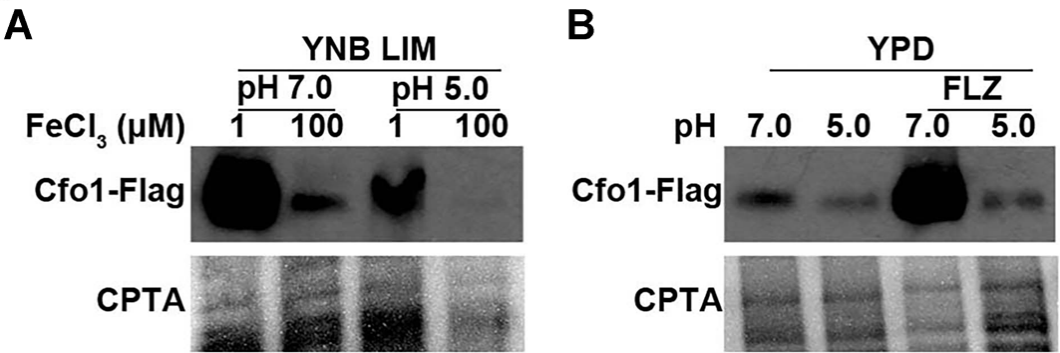
Expression of the Cfo1 protein at different pH in the presence of iron or fluconazole. (**A**) The *C. neoformans* strain carrying the Cfo1 protein fused with FLAG was cultured in LIM (pH 7.0 or 5.0) containing 1 μM or 100 μM FeCl_3_. The levels of the Cfo1–FLAG protein were evaluated by western blot analysis using anti-FLAG antibody. (**B**) The *C. neoformans* strain carrying the Cfo1 protein fused with FLAG was grown in YPD at pH 7.0 or 5.0 with or without 10 μg/ml of fluconazole. The levels of the Cfo1–FLAG protein were evaluated by western blot analysis using anti-FLAG antibody. Copper phthalocyanine–3,4',4'',4'''-tetrasulfonic acid tetrasodium (CPTA)-stained images indicate equal loading of each sample. All experiments were performed three times; the representative results are presented.

Based on the results of the growth assay, we investigated whether the expression of the high-affinity reductive iron uptake system is influenced by environmental pH. We generated a strain expressing the FLAG-tagged Cfo1 protein and evaluated the levels of the fusion protein in cells grown in low- and high-iron media at different pH conditions. The western blot analysis revealed that the expression of Cfo1–FLAG was higher in the cells grown in the low-iron medium than in those grown in the high-iron medium at pH 7.0, confirming the requirement for Cfo1 in the high-affinity reductive iron uptake system in *C. neoformans* (Fig. 2A). However, the expression of Cfo1–FLAG was significantly lower in both low- and high-iron media at pH 5.0 than at pH 7.0, suggesting that acidic pH reduces the expression of Cfo1 and that the requirement of the high-affinity reductive iron uptake system is minimal in *C. neoformans* under acidic conditions.

As fluconazole susceptibility was influenced by both environmental pH levels and the iron uptake system in *C. neoformans*, we also investigated the expression of Cfo1–FLAG in cells grown in the presence or absence of fluconazole. At pH 7.0, the expression of the protein was notably higher in the cells grown in the presence of fluconazole, while a moderate level of Cfo1–FLAG was detected in those grown in the absence of the drug, indicating that Cfo1 is induced under azole antifungal stress at neutral pH. This finding suggests that *C. neoformans* requires a high amount of iron for survival against fluconazole at pH 7.0 and that the high-affinity reductive iron uptake system composed of Cfo1 is the major pathway responsible for iron transport (Fig. 2B). In contrast, the expression of Cfo1–FLAG was similar between the cells grown with or without fluconazole at pH 5.0. These results suggest that fluconazole susceptibility and the mechanism of iron uptake are independent of the high-affinity reductive iron uptake system, further supporting the aforementioned results of the fluconazole susceptibility test performed using mutant strains lacking *CFO1*.

### Acidic pH Increases Iron Accumulation in *C. neoformans* Cells

**Fig. 3:**
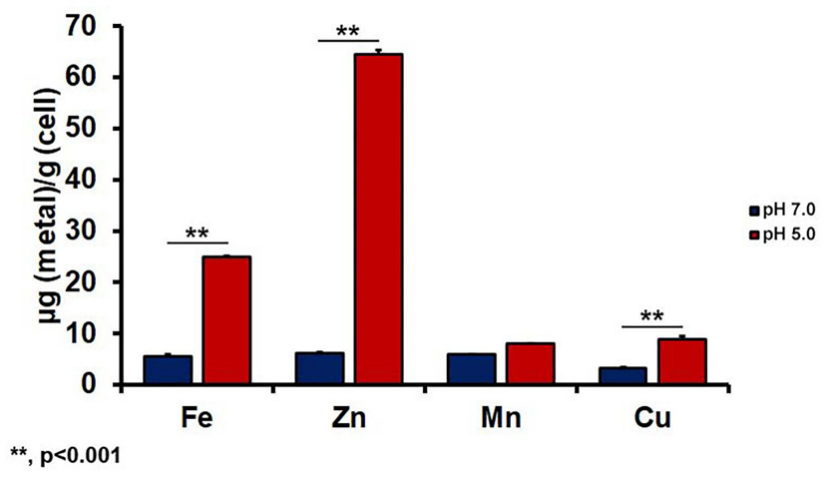
Intracellular metal contents in cells grown at different pH. The cells were cultured in LIM (pH 7.0 or 5.0). Intracellular concentrations of Fe, Zn, Mn, and Cu were determined by ICP analysis. Values indicate weight per total cell mass and are averages from three independent experiments with standard deviations.

We next measured intracellular iron levels in *C. neoformans* cells grown at different pH conditions. We also measured the intracellular levels of other essential metals, including zinc, copper, and manganese. The results demonstrated that cells grown at pH 5.0 had higher intracellular iron levels, suggesting that an acidic environment enhances iron accumulation in this fungus (Fig. 3). Based on our findings mentioned above, the increase in iron accumulation in cells grown under acidic conditions is likely mediated by the iron uptake system independent of the high-affinity reductive iron uptake system. Moreover, these findings suggest that such an iron uptake system transports extracellular iron to the cells more efficiently at acidic pH than at neutral pH. We also noted increased intracellular accumulation of zinc and copper in *C. neoformans* cells, suggesting that different uptake systems or efficiencies mediate the transport of these metals in *C. neoformans* under different pH conditions. As an exception, the intracellular manganese levels were similar in cells grown at pH 5.0 and those grown at pH 7.0, implying that manganese uptake is less affected by environmental pH.

### *C. neoformans* Synthesizes More Heme and Ergosterol at Acidic pH

**Fig. 4:**
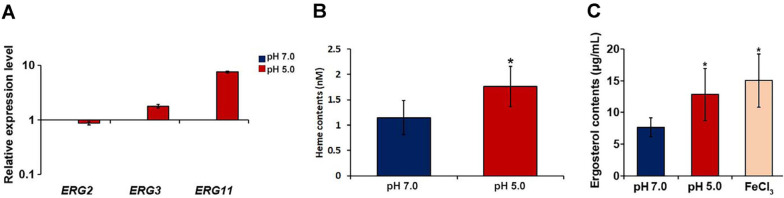
Expression of *ERG* genes and intracellular levels of heme and ergosterol in *C. neoformans* cells grown under different pH conditions. (**A**) The *C. neoformans* WT strain was grown at pH 7.0 or 5.0, and expression levels of *ERG2*, *ERG3*, and *ERG11* were measured by q-PCR. The data are presented as the expression of each gene in the cells grown at pH 5.0 relative to the value obtained from the cells grown at pH 7.0 (set at 1). Values are averages from three replicates, and bars represent standard deviations. (**B**) The *C. neoformans* WT strain was grown at pH 7.0 or 5.0, and intracellular heme concentrations were determined and normalized by cell biomass (wet weight). Results are averages from three replicates, and bars represent standard deviations. (**C**) The *C. neoformans* WT strain was grown at pH 7.0 or 5.0, and intracellular ergosterol concentrations in 1 ml cell suspension containing 1 × 10^8^ fungal cells were determined. Results are averages from three replicates, and bars represent standard deviations.

Ergosterol is a major constituent of fungal cell membranes; its biosynthesis pathway is composed of several Erg enzymes and is a direct target of azole antifungal drugs, including fluconazole [34]. Therefore, we investigated changes in the expression of representative *ERG* genes, such as *ERG2*, *ERG3*, and *ERG11*, at different pH. *ERG11* was of particular interest because it encodes the Erg11 protein, which is a heme‐containing P450 enzyme (lanosterol 14α‐demethylase) and a direct target of fluconazole [35]. Our qPCR results revealed that the expression levels of *ERG3* and *ERG11* were higher at pH 5.0 than at pH 7.0 (Fig. 4A). Notably, the transcript levels of *ERG11* were considerably higher at pH 5.0 than at pH 7.0, indicating that the expression of this gene was highly influenced by external pH.

As mentioned above, Erg11 possesses heme as a cofactor. The upregulation of *ERG11* was correlated with higher heme availability in the cells [36, 37]. We measured intracellular heme levels in *C. neoformans* cells grown at different pH and found that intracellular heme levels were significantly higher at pH 5.0 than at pH 7.0 (Fig. 4B). Heme biosynthesis requires iron as the central atom in the porphyrin ring [38]. Therefore, these results suggest that more abundant intracellular iron in *C. neoformans* at acidic pH increases the production of heme in the fungus. These findings corroborate our findings mentioned above. That is, increased expression of *ERG* genes and increased intracellular heme levels under acidic conditions appear to increase ergosterol synthesis in *C. neoformans*. We also observed higher ergosterol content in cells at pH 5.0 than at pH 7.0 (Fig. 4C).

**Fig. 5:**
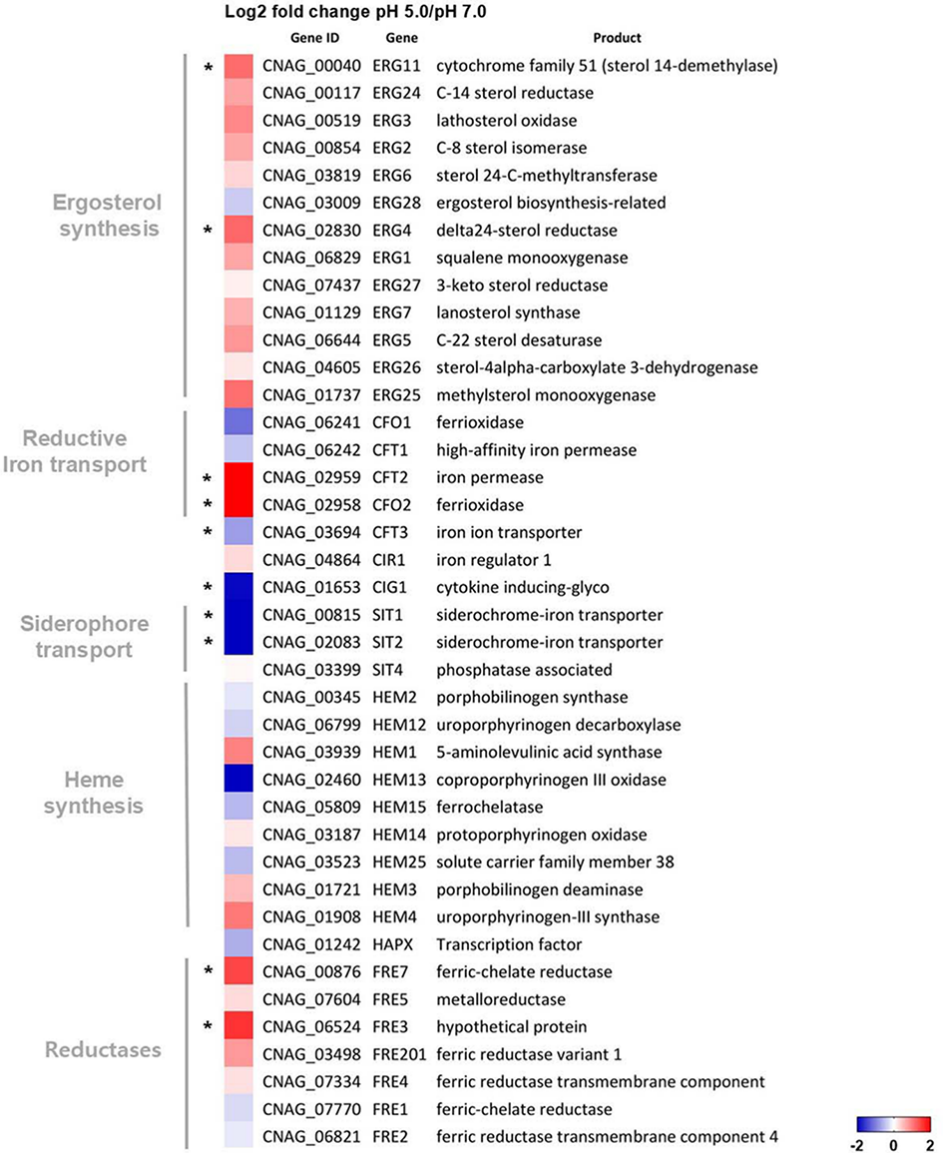
Differentially expressed genes in *C. neoformans* cells grown at pH 5.0 compared with those in cells grown at pH 7.0. Genes encoding proteins involved in ergosterol synthesis, reductive iron transport, siderophore transport, heme synthesis, and reductases were selected and are shown in the heat-map. The transcriptome results were obtained from two biological replicates. Asterisks indicate data with statistical significance (*p* < 0.05).

To further support our findings, we performed transcriptome analysis via RNA sequencing to compare differential gene expression levels between *C. neoformans* cells grown at pH 5.0 and 7.0. As shown in Fig. 5, the transcript levels of genes involved in ergosterol biosynthesis and heme biosynthesis in *C. neoformans* cells grown at pH 5.0 were generally higher than those in cells grown at pH 7.0. *C. neoformans* possesses a total of eight *FRE* genes encoding putative ferric reductases [39]. Among these, the transcript levels of *FRE3* and *FRE7* were significantly higher at pH 5.0 than at pH 7.0, indicating that the cell possesses relatively higher reductase activity under acidic conditions. Moreover, the expression levels of siderophore transporter genes (*SIT*) and genes involved in the high-affinity reductive iron uptake system (*CFO1* and *CFT1*) were lower in *C. neoformans* cells grown at pH 5.0 than in those grown at pH 7.0, confirming our findings mentioned above.

Taken together, *C. neoformans* possesses higher intracellular iron levels at acidic pH than at neutral pH. This increases the production of intracellular heme and upregulates the expression of *ERG* genes, including *ERG11*, thereby increasing ergosterol synthesis. Our findings highlight that acidic environments increase iron transport in *C. neoformans* independent of the Cfo1- and Cft1-mediated high-affinity reductive iron uptake system, thereby allowing the fungal cells to synthesize more heme and ergosterol, enhancing membrane composition, and improving tolerance to fluconazole.

## Discussion

In this study, we investigated the influence of environmental pH on the susceptibility of *C. neoformans* to fluconazole, focusing on how pH affects iron uptake and consequently impacts *ERG* gene expression, ergosterol production, and heme biosynthesis. We found that *C. neoformans* exhibits lower susceptibility to fluconazole at acidic pH than at neutral pH. These results suggest that an alternative iron uptake mechanism operates independently of the high-affinity reductive iron uptake system composed of Cfo1 and Cft1 under acidic conditions. This alternative iron uptake pathway is responsible for increased intracellular iron levels, heme levels, *ERG* gene expression, and ergosterol biosynthesis under acidic environments, all of which collectively alter the composition of the fungal cell membrane. Consequently, fungal cells grown at acidic pH levels exhibit higher tolerance to fluconazole than those grown at neutral pH.

Our initial observation that *C. neoformans* is less susceptible to fluconazole at acidic pH is well supported by the finding of Carlson *et al*. that environmental factors may significantly influence the efficacy of antifungal drugs, including fluconazole [2]. The mechanism of action of fluconazole involves the inhibition of the activity of Erg11, which is essential for ergosterol synthesis [35]. Iron is crucial for the function of Erg11 because the enzyme requires heme as a cofactor. Ergosterol is an essential constituent of the fungal cell membrane, maintaining its integrity and fluidity. We observed that the susceptibility of the mutant lacking *CFO1* or *CFT1* to fluconazole was similar to that of the WT strain at pH 5.0; however, the same mutants showed increased susceptibility to the drug at pH 7.0. These data indicate that *C. neoformans* utilizes an alternative iron uptake pathway that is independent of the high-affinity reductive iron uptake system to maintain ergosterol biosynthesis under acidic conditions. The reduced expression of the Cfo1 protein noted at pH 5.0 further suggests a shift in iron uptake mechanisms under acidic conditions. This observation suggests that the high-affinity reductive iron uptake system, which is required for iron acquisition at neutral pH, is bypassed or less crucial in *C. neoformans* in acidic environments.

Although our study provides valuable insights into the mechanisms underlying pH-dependent fluconazole susceptibility, we could not experimentally identify and characterize which transport system is responsible for iron uptake in *C. neoformans* at acidic pH. However, our results suggest that such an alternative iron uptake system involves Fe^2+^ transport. Using the mutant lacking *CFO1* or *CFT1*, we demonstrated that the high-affinity reductive iron uptake system is unnecessary for iron uptake in *C. neoformans* at pH 5.0. Moreover, our transcriptome analysis revealed significantly upregulated expression of *FRE* genes encoding ferric reductases, suggesting that the production of Fe^2+^ on the surface of *C. neoformans* cells increases under acidic conditions. Saikia *et al*. [39] found a total of eight *FRE* genes in *C. neoformans*. They reported that the deletion of *FRE4* reduced melanin production and that the mutant lacking *FRE2* exhibited growth deficiency in medium containing heme or transferrin as the sole iron source and reduced virulence in a mouse model of cryptococcosis. However, they did not clearly characterize the functions of other *FRE* genes, possibly due to their redundant roles. Therefore, future research should assess fluconazole susceptibility and iron uptake of mutants lacking *FRE* genes, especially under acidic conditions.

In *Saccharomyces cerevisiae*, iron uptake systems, including the high-affinity reductive iron transport system, have been extensively studied [40, 41]. However, little is known regarding iron uptake in acidic environments. In a previous study, no alteration was noted in the expression of Fet3 and Ftr1, which are homologs of *C. neoformans* Cfo1 and Cft1, respectively; however, the expression of the cell surface reductases Fre1 and Fre2 was highly upregulated in *S. cerevisiae* under acidic conditions, implying increased production of Fe^2+^ [42]. In contrast, studies have identified and characterized Fe^2+^-specific transport systems in various gram-negative bacteria, such as *Escherichia coli*, *Bordetella pertussis*, and *B. bronchiseptica*. These bacteria possess the Feo and FtrABCD systems, which are responsible for Fe^2+^ uptake under acidic conditions [43, 44].

We found that the expression of *ERG* genes, particularly *ERG11*, was upregulated under acidic conditions. This increased expression of *ERG11*, a direct target of fluconazole, likely increases the concentration of the target enzyme and overcomes the inhibitory effects of fluconazole. Furthermore, increased *ERG11* expression is well correlated with higher intracellular heme levels, as Erg11 is a heme-containing enzyme [45, 46]. These findings suggest that *C. neoformans* compensates for the inhibition of Erg11 by producing greater amounts of the enzyme and ensuring an adequate supply of its cofactor, heme. Increased iron uptake, heme biosynthesis, and *ERG* gene expression eventually increase the production of ergosterol at acidic pH. This increased ergosterol production alters the cell membrane composition of *C. neoformans* and may prevent fungal cells from the effects of fluconazole, such as interference with membrane synthesis and disruption of membrane integrity, thereby conferring resistance to fluconazole under acidic conditions. This finding is consistent with previous findings that increased ergosterol levels can mediate azole resistance in various fungal species [47-49].

Our observation that pH influences the susceptibility of *C. neoformans* to fluconazole has significant implications for the pathogenesis and treatment of cryptococcosis. *C. neoformans* commonly infects the lungs and CNS, where environmental pH may vary depending on the host microenvironment and the presence of inflammation [50-52]. Several studies have suggested that inflamed tissues normally exhibit acidic microenvironments because of increased metabolic activity and lactic acid production [53-55]. Furthermore, studies have revealed that a phagolysosome within host phagocytes is an acidic cellular compartment, suggesting that the intracellular nature of *C. neoformans* should also be considered to play a role in reducing its susceptibility to fluconazole under acidic environments [24, 56]. Together, our data suggest that *C. neoformans* adapts to acidic environments by increasing iron uptake, heme biosynthesis, and ergosterol production, which may contribute to its persistence and virulence in host niches.

In conclusion, our findings highlight the importance of considering environmental factors in the development and optimization of antifungal treatment strategies for cryptococcosis. Further research is needed to fully elucidate the complex interplay between pH, iron homeostasis, ergosterol biosynthesis, and drug susceptibility in *C. neoformans*. Identification and characterization of alternative iron uptake mechanisms at acidic pH could also help develop a novel antifungal drug target and understand the adaptive strategies employed by *C. neoformans* in acidic environments.

## Supplemental Materials

Supplementary data for this paper are available on-line only at http://jmb.or.kr.
