# Supplementary material for: Acidic pH Reduces Fluconazole Susceptibility in Cryptococcus neoformans by Altering Iron Uptake and Enhancing Ergosterol Biosynthesis
Source: J Microbiol Biotechnol. 2025 May 27;35:e2504007. doi: 10.4014/jmb.2504.04007 (PMC12149401; doi:10.4014/jmb.2504.04007)
Supplement: Supplementary file 1 [file jmb-35-e2504007-supple.pdf]

Supplementary Materials

Table S1. Primers used in this study.

Primers	Sequence
Cfo1_BamHI_F	TCGCTA GGATCC ATGTACTCGACTACAGCTCTGC
Cfo1_BamHI_R	AGGAATGGATCCTTACACTTTGACATCAGTCTTCAA
ERG2_qPCR_F	ACCTCTCAACCCTACCATCC
ERG2_qPCR_R	ATCAGTCAGCTTTCCGACC
ERG3_qPCR_F	TCTTCCCATTCATCTTCCCTC
ERG3_qPCR_R	TCCCAGCTTCATCTATCAGTC
ERG11_qPCR_F	TGGAGAACAGGAGAAAGGG
ERG11_qPCR_R	CAAGAAGAAGTAGCAGAGGAAG
